# Taiwanese parents’ perspectives on young children’s use of information communication technology

**DOI:** 10.3389/fpsyg.2023.1248863

**Published:** 2023-09-19

**Authors:** Yi Fang Luo, Shu Ching Yang, Kun Yi Chou, Hsin Tien Lee

**Affiliations:** ^1^Graduate School of Human Sexuality, Shu-Te University, Kaohsiung City, Taiwan; ^2^Institute of Education, National Sun Yat-sen University, Kaohsiung City, Taiwan

**Keywords:** technology application, mobile device, young children, children’s ICT use, parents’ perceptions of children’s ICT usage

## Abstract

**Introduction:**

How parents think and feel about their children’s use of technology can influence how their kids behave online. The family’s socioeconomic status (SES) may also affect this influence. In light of this, this research emphasizes the need for more investigation into parental attitudes and the role of SES in shaping how children consume media.

**Methods:**

This study surveyed 629 Taiwanese parents to explore their attitudes toward their young children’s use of information communication technology (ICT), usage patterns, and the interplay with socioeconomic status.

**Results:**

The findings revealed a significant disconnect: although approximately 50% of parents considered above six years old to be a suitable age for children to start ICT, over 80% of children had already engaged with ICT before that age, indicating a large disparity between parental expectations and actual initiation. Furthermore, parents highlighted “learning interest” and “various content” as the most positive impacts of children’s ICT use, while “addiction and overreliance” emerged as their primary concern. Notably, parents, as a whole, tended to perceive their child’s ICT use more negative than positively, with fathers displaying greater acceptance of negative viewpoints than mothers. Parental attitudes toward children’s ICT use were categorized into five clusters, ranging from balanced and optimistic views to value emphasis, conservatism, and negative doubts. This classification underscores the intricate and multifaceted nature of parental perspectives, encompassing both positive and negative outlooks on children’s ICT utilization.

**Discussion:**

The findings underscore the nuanced character of parents’ attitudes toward technology, shaped by the intricacies and challenges posed by the digital era. These insights emphasize that parental attitudes go beyond a simplistic positive-negative divide, reflecting a comprehensive response to the opportunities and complexities inherent in the digital age.

## Introduction

Online video viewing dominates children’s screen time. According to a recent report by The Common Sense Census (Rideout and Robb, 2020), approximately 50% of children aged 2–4 and more than two-thirds of children aged 5–8 have their own tablet or smartphone. This also means that ICT has become an integral part of children’s lives and is considered a part of early childhood development (Epstein, 2015; Jack and Higgins, 2019). The COVID-19 pandemic and subsequent lockdowns further accelerated this trend (Bergmann et al., 2022). A survey report pointed out that children aged 0–2 will spend 39 min a day watching online videos on platforms such as YouTube and TikTok in 2020, up from 19 min in 2017 (Rideout and Robb, 2020). In Kuo (2022) report on children aged 3 to 6 in Taipei City (785 participants) found that 78.5% used communication technology products.

The utilization of ICT by children has witnessed significant growth in recent years, yet debate persists about its suitability in the preschool context (Common Sense Media, 2013; Lin, 2013). In this discourse, parents emerge as the primary guardians, tasked with shielding their children from inappropriate media exposure. Balancing the role of guiding and supervising their children’s digital explorations while enabling unrestricted exploration has become their responsibility (Vandewater et al., 2005; Dias et al., 2016). Hence, the principal objective of this study is to gain insights into parental perspectives concerning children’s engagement with ICT, focusing on computers and smartphones.

The thoughts and attitudes parents hold regarding their children’s interaction with ICT sway their offspring’s online conduct (Gür and Türel, 2022), potentially modulated by the socioeconomic status (SES) of the family unit (Sun et al., 2021). Against this backdrop, this study underscores the imperative for further exploration into parental attitudes and the interplay of SES in shaping children’s media consumption patterns. The researchers comprehensively explore young children’s ICT usage and parents’ perspectives on integration and analyze positive and negative viewpoints as well as differing parental attitudes toward positive and negative ICT use. In addition, we reveal the specific profiles of parents’ viewpoints pertaining to children’s ICT engagement. Concurrently, the study investigates the intricate relationship linking SES and children’s media usage. By untangling these multifaceted factors, this research can offer invaluable insights to policymakers, empowering them to craft effective strategies that cultivate secure and appropriate media engagement practices among young children. In turn, this fosters a culture of responsible and conscientious media use within Taiwanese families.

## Literature review

### Pros and cons of using ICT for young children

The use of ICT among young children has both advantages and disadvantages. Advocates of ICT use in early childhood education argue that technology, when utilized appropriately, can offer numerous benefits. These include providing interactive and engaging learning experiences, fostering early literacy and numeracy skills, enhancing problem-solving abilities, and nurturing creativity and curiosity (Blackwell et al., 2014; Ley et al., 2014; Sung et al., 2016). Moreover, ICT can expose children to diverse cultures, languages, and perspectives through multimedia content, enriching their worldview. However, critics raise valid concerns regarding potential risks associated with early and excessive ICT use. Such risks include sedentary behavior, diminished face-to-face social interactions, disruptions in sleep patterns, and attention-related challenges (McOptom, 2017). Moreover, excessive ICT exposure might hinder the development of self-regulation skills, thus impeding empathetic understanding, imaginative thinking, in-depth reading, and constructive reasoning abilities (DeLoatch, 2015). Excessive screen time may also encroach upon other vital activities, such as outdoor play, imaginative play, and interactions with peers and caregivers, potentially contributing to an increased risk of early age obesity (Chaudron et al., 2015; Haughton et al., 2015).

The aforementioned discussion underscores the fact that the use of ICT by children is not strictly advantageous or disadvantageous; it entails a nuanced trade-off between the two. Parents, as primary influencers of their children’s beliefs, attitudes, and behaviors, are encouraged to exercise caution in facilitating their children’s mindful and appropriate use of technology. By doing so, parents can help children maximize the benefits of technological opportunities while mitigating potential downsides (Gür and Türel, 2022). However, achieving this balance poses a challenge.

A study by Golden et al. (2020) revealed that a significant number of parents aspire to strike a better equilibrium between smartphone usage and traditional activities with their infants. Despite this aspiration, many parents struggle to achieve such a balance. This may indicate a degree of uncertainty among parents about how ICT use can positively impact their children. The extent of this disconnect remains largely unexplored, prompting us to pose the following research question:

RQ: Are there discernible differences in parents’ perceptions of children’s ICT usage?

The utilization of ICT by young children is often driven by the perceived educational benefits it offers. Within the educational context, Chinese culture has distinct characteristics, with fathers often assuming the role of stern disciplinarians, while mothers foster close and affectionate relationships with their children (Lim and Soon, 2010; Xing et al., 2019). Although the perspective of fathers is not considered in this context, research by Lim and Soon (2010) revealed that mothers frequently perceive themselves as having less ICT knowledge and skills compared to both their children and husbands. Furthermore, mothers occasionally believe that their spouses consider them overly restrictive in regulating their children’s ICT use, resulting in conflicts between spouses. Building on these observations, this study posits the following hypothesis:

H_1_: Distinct positive and negative perspectives regarding their children’s ICT use exist between mothers and fathers.

### Parental influence on young children’s ICT use

Parents’ vigilant awareness of their young children’s ICT utilization propels their offspring to optimize the advantages offered by technological opportunities (Ramírez-Rueda et al., 2021; Gür and Türel, 2022). The study conducted by Gür and Türel (2022) revealed that parents’ attitudes toward ICT usage among 12- to 14-year-olds leaned toward control and constraint due to concerns about the potential adverse impacts of ICT use. Despite this apprehension, these parents acknowledged the educational utility of ICT tools. The research by Gür and Türel employed a survey methodology to probe into parental attitudes concerning children’s engagement with ICT. This quantitative approach was supplemented by interviews involving 13 parents, aimed at capturing deeper insights into their perspectives on children’s ICT utilization and discerning the rationale behind their inclination to restrict its usage. An important limitation of Gür and Türel’s study arises from the relatively small number of parents interviewed, which represents only a fraction of those surveyed through the questionnaire.

To further solidify the direct correlation between parental perceptions of children’s ICT use and the subsequent imposition of restrictions on such usage, the present study adopted an altered research methodology and statistical approach. As a response to the aforementioned limitation, this study posits the following hypotheses:

H_2_: Parents’ positive and negative views regarding their children’s ICT use predict the level of parental regulation over young children’s ICT use.

Healy (2000) emphasized that computer proficiency stands as a fundamental technological skill that parents aspire for their children to cultivate. However, whether situated within the confines of home or the structured environment of school, children’s access to ICT resources remains disparate. A discernible discrepancy surfaces, wherein children hailing from families with high SES end up enjoying relatively enhanced access to ICT (Saçkes et al., 2011). This assertion gains further credence from Chen (2008) investigative work, which underscored how a family’s SES exerts a tangible influence over the acquisition and utilization of ICT hardware and software. As a consequence, it shapes the extent and manner through which young children are introduced to and educated about ICT.

However, the sway of family SES is not confined solely to determining young children’s ICT access; it extends its grasp to encompass parents’ apprehensions regarding their children’s ICT engagement (Mollborn et al., 2022). This is evidenced by the findings of Livingstone (2007) study, which revealed that parents endowed with elevated SES more frequently take on the role of regulating their children’s interactions with television, videos, and other forms of media than parents with lower SES. Vandewater et al. (2005) further substantiated this by highlighting that parents boasting higher household income and educational attainment levels are more inclined to oversee their children’s television viewing duration and the nature of programs they consume. Children with lower parental education spend nearly 3 h using electronic devices on average, while those with higher parental education spend approximately 1.5 h (Rideout, 2017). Yang and Chang (2021) also found that as electronic products become more affordable, the ownership of devices has increased in low-income families. Studies abroad show that children from low-income families spend more time on electronic devices than their high-income counterparts, highlighting the growing disparity. Additionally, Vandewater et al. (2005) identified a correlation between parental attitudes toward television watching and the resultant behavior of young children. Notably, a negative parental stance toward television was associated with more stringent regulation over their children’s television-watching habits, ultimately translating into reduced screen time.

Building upon this backdrop, the present study proposes the following hypothesis:

H_2_: Family SES (education levels and monthly household income) predicts children’s ICT use, including the appropriate age of initiation, actual age of initiation, frequency of usage, and parental regulation over young children’s ICT use.

## Materials and methods

### Participants

Conducted in southern Taiwan, the study engaged parents with children under 8 years old. The distribution of questionnaires occurred via kindergartens and elementary schools, yielding 675 responses. After excluding 46 incomplete “Parental attitudes toward young children’s ICT use scale” questionnaires, 629 complete responses remained (Table 1). Among the respondents, 77.74% (*n* = 489) were mothers, while 21.46% (*n* = 135) were fathers.

**TABLE 1:** Background characteristicsof the study participants (*N* = 629).

Characteristics	*n*	%	Characteristics	*n*	%
**Role**			**Age**		
Mother	489	77.74	< 31 years	30	4.77
Father	135	21.46	31–35 years	194	30.84
No answer	5	0.79	36–40 years	250	39.75
			>40 years	146	23.21
Monthly household income			No answer	9	1.43
NTD 50K or less	147	23.37	Education		
NTD 50K–100K	287	45.63	<High school diploma	124	19.71
NTD100K–150K	84	13.35	Specialist qualification	154	24.48
NTD150K–200K	23	3.66	University degree	259	41.18
NTD 200K or more	51	8.11	>Graduate school	84	13.35
No answer	37	5.88	No answer	8	1.27

Regarding the age distribution of the parents, the majority were in the 36–40-year-old age group (*n* = 250, 39.75%), followed by 31–35 years old (*n* = 194, 30.84%), 41 years or older (*n* = 146, 23.21%), and 30 years or younger (*n* = 30, 4.77%). In terms of education, the highest proportion of parents held a university degree (*n* = 259, 41.18%), followed by a specialist qualification (*n* = 154, 24.48%), a high school diploma or below (*n* = 124, 19.71%), and a graduate degree or above (*n* = 84, 13.35%). Regarding monthly household income, the majority of parents reported earning NTD 50K–100K (*n* = 287, 45.63%), followed by NTD 50K or lower (*n* = 147, 23.37%), NTD 100K–150K (*n* = 84, 13.35%), NTD 200K or more (*n* = 51, 8.11%), and NTD 150K–200K (*n* = 23, 3.66%).

### Procedure

This study focused on parents with children aged 8 or below residing in households situated in southern Taiwan. To gather data, we employed a convenience sampling technique and distributed paper-and-pencil questionnaires through schools. We contacted 16 elementary schools affiliated with kindergartens to assist in distributing the questionnaires to parents. Before dissemination, instructors provided clear information to parents about the research’s purpose, procedures, and requirements. Participants were assured that their involvement was completely voluntary, anonymous, and treated with the utmost confidentiality. They had the right to decline participation at any time. Informed consent information was presented on the first page of the questionnaire, and respondents proceeded with filling out the questionnaire only after agreeing to participate. To maintain anonymity, participants were instructed not to include any personal information on the questionnaire and were responsible for submitting it themselves. As a token of appreciation, participants received a gift along with the questionnaire, even if they later decided to withdraw from the investigation.

### Instruments

#### Background information

The background information questions aimed to capture parental demographics such as age, gender, education, and monthly household income. Parental education and household income serve as indicators of SES.

#### The status of young children’s ICT use

This part aimed to understand young children’s ICT usage at home. Participants were asked about their children’s experience with computers or smartphones and TV-watching habits (used for comparison). For children with ICT experience, additional inquiries included age at first use, frequency of use, parental participation, and main reasons for allowing ICT use (e.g., leisure, cognitive learning, rewards, appeasement, and occupying children during parental work).

#### Parental regulations of young children’s ICT use

For all participants, we examined their views on regulating their children’s ICT use with two items: the appropriate age for computer/smartphone use and the parents’ attitudes toward regulating their children’s use. Responses were rated on a scale of 1 to 3: 1 = no limit and can always use, 2 = limited use, and 3 = complete ban. Higher scores indicate a more rigid parental attitude.

#### Parental attitudes toward young children’s ICT use scale

To measure parental attitudes toward young children’s ICT use (e.g., computers, smartphones), the researchers, based on previous studies, compiled the Parental Attitudes toward Young Children’s ICT Use Scale; moreover, its content and validity were also verified by experts. This five-point Likert scale ranged from “disagree completely” (1) to “agree completely” (5). Through exploratory factor analysis (principal axis factoring extraction; eigenvalue >1), a two-dimensional structure was derived. The Kaiser–Meyer–Olkin (KMO) test yielded a value of 0.946 (χ2 = 12345.50, *p* < 0.001), with a significant Bartlett’s sphericity test result (*p* < 0.05). The first dimension, “positive effects,” encompassed 13 items, while the second dimension, “negative effects,” contained 10 items. The combined variance explained was 65.57%. Both the “positive effects” and “negative effects” dimensions exhibited strong internal reliability scores of 0.95.

#### Data analysis

The study’s data analysis encompassed several steps. Initially, we employed SPSS Statistics version 20.0 to construct descriptive statistics, facilitating an understanding of both young children’s ICT use and parental attitudes. Subsequently, an exploratory factor analysis was executed to assess subscale validity and ascertain the psychometric properties of the scale. Two types of *t-*tests were utilized with a paired-sample *t-*test to analyze differences in parents’ views on positive and negative aspects of children’s ICT use and an independent *t*-test to explore potential disparities between mothers and fathers in their attitudes toward these dimensions of their children’s ICT usage. Furthermore, a k-means clustering analysis was conducted to identify distinct groups of parents based on their patterns of positive and negative conceptions regarding children’s ICT use. The analysis involved hierarchical clustering utilizing Ward’s method to preliminarily determine clusters, followed by K-means clustering with varying cluster counts. This approach was validated through discriminant analysis. Variance analysis was also applied to validate the clustering results and assess the significance of differences between clusters. Finally, a regression analysis was conducted to probe the relationships among family SES, incorporating factors such as education level and monthly household income, and various dimensions of young children’s ICT use. This analysis provided insights into the associations among family SES variables and ICT use status, parental regulations, and attitudes.

## Results

### The status of young children’s ICT use

Table 2 outlines the current status of young children’s ICT use, revealing that 50.24% of parents (*n* = 316) mentioned that their children used computers, and 33.07% (*n* = 208) reported smartphone usage among their children younger than eight.

**TABLE 2:** The status of young children’s ICT use.

	Computer (*n* = 336)		Smartphone (*n* = 219)	
	***n***	**%**	***n***	**%**
**Age at first use**				
Younger than 3 years old	37	11.01	25	12.02
3 to 5 years old	242	72.02	161	77.40
6 to 8 years old	37	11.01	22	10.58
No answer	20	5.95	11	5.29
**Frequency of use**				
Rarely use	155	46.13	79	37.98
Occasionally use	122	36.31	92	44.23
Often use	51	15.18	42	20.19
No answer	8	2.38	6	2.88

Of the 336 parents whose children had experience with computers, 11.01% stated that their children started before the age of two. However, the majority (72.02%) reported that their children began computer use between ages three and five, coinciding with the kindergarten phase. Regarding computer use frequency, 15.18% of parents reported “often,” 36.31% “occasionally,” and 46.13% “rarely.” Likewise, among 219 parents whose children used smartphones, 12.02% said their children started before age two. The majority (77.40%) reported that their children started smartphone use between ages three and five, similar to computer use. Regarding frequency, 20.19% of parents reported “often,” 44.23% “occasionally,” and 37.98% “rarely.”

Among the 396 parents with children experienced in using computers or smartphones, 41.67% (*n* = 165) allowed ICT use to support cognitive learning. Other purposes included leisure and entertainment (*n* = 141, 35.61%), rewards (*n* = 27, 6.82%), and appeasement/occupying children during parental work (14 individuals, or 3.54% for each category). However, 8.84% (*n* = 35) of parents did not respond. Regarding parental companionship during ICT use, 40.66% (*n* = 161) always accompanied their children, 34.09% (*n* = 135) often accompanied them, 19.44% (*n* = 77) sometimes accompanied them, 3.79% (*n* = 15) rarely accompanied them, and 1.52% (*n* = 6) did not accompany them at all. Finally, 0.51% (*n* = 2) of parents did not provide a response.

### Parental regulations of young children’s ICT use

Regarding the appropriate age for children to start using computers, Table 3 shows that 48.81% of parents considered 6 years old or later as a suitable age to start using a computer, and 58.35% reported the same age for smartphones, with 24.32% of parents selecting the age range of three to five for computers and 13.51% for smartphones. The highest percentage was for the age range of three to five for computers (17.17%) and smartphones (13.51%). A similar pattern was observed for both computer and smartphone use.

**TABLE 3:** Appropriate age for ICT use.

AGE	Computer				Smartphone			
	**Whole (*n* = 629)**		**Experienced (*n* = 336)**		**Whole (*n* = 629)**		**Experienced (*n* = 219)**	
	***n***	**%**	***n***	**%**	***n***	**%**	***n***	**%**
<3 years old	108	17.17	38	11.31	85	13.51	23	10.50
3 to 5 years old	153	24.32	116	34.52	85	13.51	60	27.40
>6 years	307	48.81	163	48.51	367	58.35	117	53.42
No answer	61	9.70	19	5.65	92	14.63	19	8.68

Among 336 parents with children experienced in using computers, 48.51% reported that 6 years old or later was suitable, and for 219 parents with children experienced in using smartphones, 53.42% indicated 6 years old or later to be the appropriate age. However, there was a discrepancy between their ideals and the reality of their children’s computer use, as more than 80% of their children had used computers and smartphones before reaching the age they considered appropriate.

We surveyed 629 parents to explore their attitudes regarding regulations on young children’s ICT use, specifically computers and smartphones. For computer use, 7.00% (*n* = 44) reported unrestricted access, while 69.63% (*n* = 438) preferred limitations on time and content. A significant proportion, 22.26% (*n* = 140), favored a complete ban, and 1.11% (*n* = 7) did not respond.

Regarding smartphone use, 6.52% (*n* = 41) supported unrestricted access, and 50.56% (*n* = 318) preferred limitations on time and content. A substantial number, 38.95% (*n* = 245), opted for a complete ban, and 3.97% (*n* = 25) did not provide a response. Parents appear to set stricter restrictions on smartphone use than on computer use.

### Parental attitudes toward young children’s ICT use

Table 4 reveals interesting insights into parental attitudes toward young children’s ICT use, unveiling a spectrum of perspectives that elucidate both positive and negative effects. Among the positive effect items, the resounding agreement (*M* = 3.62) with the statement “21. Rich audio and video improve interest in learning” underscores a unanimous belief in the potency of multimedia to ignite children’s passion for learning. This attests to the recognition of ICT’s dynamic potential to engage and captivate young learners, hinting at the evolving landscape of education. Delving deeper, the items “2. Provide a variety of learning content (*M* = 3.50)” and “3. Diverse stimuli for learning (*M* = 3.44)” resonate as pivotal points. These findings emphasize not only a broad acceptance of ICT’s role in diversifying learning experiences but also a more nuanced understanding of its capacity to stimulate various facets of cognitive development.

**TABLE 4:** Descriptive statistics of the parental attitudes toward young children’s ICT use.

No.	Item	Response %					*M*	*SD*	*Rank*
		**(1)**	**(2)**	**(3)**	**(4)**	**(5)**			
**Positive effect**									
1	Help children think flexibly.	5.09	16.85	37.52	34.66	5.88	3.19	0.96	
2	Provide a variety of learning content.	4.13	8.11	30.37	48.49	8.90	3.50	0.92	2
3	Provide diverse stimuli for learning.	4.13	10.97	30.05	46.74	8.11	3.44	0.94	3
4	Training the ability to react.	4.77	13.04	34.50	41.02	6.68	3.32	0.95	4
5	Contribute to the development of cognitive skills (the promotion of thinking skills and knowledge).	5.25	12.40	36.72	38.79	6.84	3.30	0.95	5
6	Contribute to language learning.	4.61	17.01	33.55	38.00	6.84	3.25	0.97	
7	Training hand-eye coordination.	7.15	14.79	34.34	38.79	4.93	3.20	0.99	
8	Contribute to more familiarity with learning content.	3.97	13.04	48.33	31.96	2.70	3.16	0.83	
9	Help children to understand the learning content.	4.61	15.90	46.26	29.41	3.82	3.12	0.88	−4
10	Have a sense of accomplishment in learning.	5.72	16.06	44.83	28.93	4.45	3.10	0.92	−3
11	Contribute to active learning.	7.00	18.28	38.79	30.37	5.56	3.09	0.99	−2
12	Help to build self-confidence in learning.	6.84	18.60	47.06	23.53	3.97	2.99	0.92	−1
13	Rich audio and video help to improve interest in learning.	3.66	8.90	21.94	52.46	13.04	3.62	0.95	1
**Negative effect**									
1	Easily lead to the phenomenon of technology-use addiction.	0.79	3.66	14.79	39.90	40.86	4.16	0.87	1
2	Develop the habit of overreliance on audio-visual learning.	0.79	3.02	20.03	46.10	30.05	4.02	0.83	2
3	Affect the development of the optic nerve.	0.64	2.86	26.07	42.29	28.14	3.94	0.84	3
4	Reduce interests in exploring the external environment.	1.11	7.31	21.46	38.79	31.32	3.92	0.96	4
5	Reduce motivation and interest in reading.	0.95	7.47	21.46	39.75	30.37	3.91	0.95	
6	Reduce creativity.	1.43	8.74	34.18	30.52	25.12	3.69	0.99	
7	Hinder the development of the ability to live independently.	1.59	10.02	34.82	33.55	20.03	3.60	0.97	−4
8	Affect the development of thinking.	1.11	10.02	37.84	32.11	18.92	3.58	0.94	−3
9	Hinder the development of relationships.	1.59	10.65	35.29	34.18	18.28	3.57	0.96	−2
10	Establish improper values.	2.38	9.86	38.79	27.19	21.78	3.56	1.01	−1

(1) = Disagree completely. (2) = Disagree. (3) = Neutral. (4) = Agree. (5) = Agree completely.

Conversely, the item “Helps build self-confidence in learning (*M* = 2.99)” showcases a more cautious stance. Here, parents appear to exhibit a degree of skepticism, hinting at a divergence of opinions regarding ICT’s potential impact on children’s self-assurance. This nuanced dichotomy within positive effects reflects the intricate tapestry of beliefs surrounding ICT’s influence on young learners.

Shifting focus to negative effects, two striking observations stand out prominently. The high agreement (*M* = 4.16) with the statement “Easily leads to the phenomenon of technology-use addiction” resonates as an alarm bell, encapsulating a widespread concern about the potential pitfalls of excessive ICT engagement. This finding captures the prevailing unease about the delicate balance between leveraging technology for learning and guarding against its potential addictive allure.

Equally important is the acknowledgment (*M* = 4.02) of the peril of “Developing the habit of overreliance on audio-visual learning.” This acknowledgment speaks volumes about the recognition of the need for a holistic educational approach that does not overemphasize one particular medium, hinting at the desire for a well-rounded learning experience. It is intriguing to observe that the concern regarding “Establishing improper values (*M* = 3.56)” is relatively lower. This suggests that while parents harbor certain reservations, there appears to be a higher level of confidence in the ability of caregivers to navigate and mitigate potential value-related challenges posed by ICT. Furthermore, the recognition of items such as “10. Hinder the development of relationships (*M* = 3.57)” and “8. Thinking (*M* = 3.57) and living independently (*M* = 3.56)” as potential issues highlight a keen awareness of the multifaceted impact of ICT on children’s holistic growth.

Moreover, the results of the paired *t*-test reveal a trend: parents, as a whole, tend to hold more negative than positive perceptions of their child’s ICT usage (*M*_P_ = 3.25, *M*_F_ = 3.80, *t* = −10.77, *p* < 0.001). This supported H_1a_. This observation indicates a prevailing sense of caution among the majority of parents regarding embracing ICT use in their children’s lives. This cautious stance suggests a desire to carefully navigate the potential impacts of technology on their children’s development and wellbeing.

### *T*-test: differences between mothers and fathers

A *t*-test was performed to analyze the differences between mothers and fathers in terms of their positive and negative attitudes toward their children’s use of ICT. The results highlighted significant differences between these two parental cohorts, particularly regarding their negative perceptions of their children’s ICT engagement, while there were no differences in their positive views. Intriguingly, fathers demonstrated a noticeably higher degree of acceptance toward negative viewpoints than mothers (*M*_M_ = 3.75, *M*_F_ = 3.52, *t* = 3.67, *p* < 0.001). H_1b_ was half supported. This finding implies that fathers may be more open or lenient in acknowledging potential drawbacks or concerns related to their children’s use of ICT. This variance in parental attitudes highlights the complexity and diversity of viewpoints within the familial context, thereby contributing to a more comprehensive understanding of the dynamics surrounding children’s interactions with ICT.

### Clustered parents based on positive and negative conceptions of children’s ICT

Using the cluster analysis method, this comprehensive study successfully categorized parents’ diverse perceptions of children’s ICT usage into distinct clusters, revealing the intricate nature of their attitudes. The analysis consisted of two main stages. In the initial stage, Ward’s method was employed for hierarchical clustering to determine the optimal number of clusters. Subsequently, in the second stage, K-means cluster analysis was conducted on parents’ perceptions of children’s ICT, using cluster numbers of 3, 4, and 5. To validate the classification outcomes, discriminant analysis was performed. The outcomes of the discriminant analysis revealed that the classification accuracy using cluster numbers 3, 4, and 5 exceeded 98%, surpassing the accuracy achieved by other cluster structures. Upon inspecting the distribution within each cluster level, it was discerned that 5 distinct clusters effectively captured various facets of parents’ perceptions of children’s ICT (Table 5). Furthermore, a variance analysis was executed using positive and negative conceptions of children’s ICT as dependent variables, while the clusters formed the independent variables. The results exhibited significant *F*-values for both positive (*F* = 551.528, *p* < 0.000) and negative (*F* = 323.935, *p* < 0.000) conceptions. This underscores the validity of the clustering outcomes.

**TABLE 5:** Cluster of parents’ views toward children’s ICT use.

Cluster	Balanced insightful	Optimistic believer	Value contradictor	Skeptical conservative	Pessimistic believer
Positive *M*=3.25	3.33 (P_R3_)	3.94 (P_R1_)	3.38 (P_R2_)	1.75 (P_R5_)	2.09 (P_R4_)
Negative *M*=3.80	3.69 (N_R3_)	2.88 (N_R4_)	4.66 (N_R1_)	2.18 (N_R5_)	4.44 (N_R2_)
P/R (R#)	P_R3_< N_R3_	P_R1_> N_R4_	P_R2_< N_R1_)	P_R5_ <N_R5_	P_R4_<N_R2_
*N* (%)	242 (38.5%)	148 (23.5%)	125 (19.9%)	9 (1.4%)	105 (16.7%)
Father	47 (34.81%)	48 (35.56%)	20 (14.81%)	1 (1.5%)	19 (14.07%)
Mother	194 (39.67%)	97 (19.84%)	104 (21.27%)	8 (1.64%)	86 (17.59%)
**Education**					
High school	45 (18.60%)	39 (26.35%)	23 (18.40%)	1 (11.11%)	14 (13.33%)
Junior college	48 (19.83%)	35 (23.65%)	44 (35.20%)	3 (33.33%)	24 (22.86%)
College	112 (46.28%)	53 (35.81%)	41 (32.80%)	2 (22.22%)	51 (48.57%)
M.A.	29 (11.98%)	15 (10.14%)	14 (11.20%)	2 (22.22%)	12 (11.43%)
Ph.D.	4 (1.65%)	5 (3.38%)	1 (0.80%)	0 (0.00%)	2 (1.90%)
No answer	4 (1.62%)	1 (0.68%)	2 (1.60%)	1 (11.11%)	2 (1.90%)
**NTD income**					
<50K	62 (25.62%)	34 (22.97%)	35 (28.00%)	1 (11.11%)	15 (14.29%)
50–100K	112 (46.28%)	68 (45.95%)	53 (42.40%)	3 (33.33%)	51 (48.57%)
100–150K	31 (12.81%)	20 (13.51%)	15 (12.00%)	2 (22.22%)	16 (15.24%)
150–200K	7 (2.89%)	10 (6.76%)	4 (3.20%)	0 (0.00%)	2 (1.90%)
>200K	4 (1.65%)	2 (1.35%)	3 (2.40%)	0 (0.00%)	5 (4.76%)
No answer	26 (10.74%)	14 (9.46%)	15 (12.00%)	3 (33.33%)	16 (15.24%)

R# indicating mean ascending ranking in each view of ICT use.

Cluster 1 -Balanced Insightful Parents: Approximately 38.5% of parents hold a balanced perspective on the effects of ICT. This cluster maintains an equilibrium viewpoint, demonstrating an insightful understanding of both the positive and negative effects of ICT.

Cluster 2–Optimistic Believer Parents: Comprising approximately 23.5% of parents, this cluster holds a strong belief in the positive impact of ICT while downplaying minor negative effects. These parents firmly endorse the idea that the benefits of ICT far outweigh potential drawbacks. Their perspective can be characterized as one of unwavering optimism, as they remain steadfast in their conviction regarding the beneficial role of ICT in education and its overall value.

Cluster 3–Value Contradictor: Consisting of approximately 19.9% of parents, this cluster distinctly emphasizes the advantageous aspects of ICT while acknowledging its potential downsides. These parents place considerable emphasis on the adverse implications that ICT can introduce to education. Their perspective can be aptly characterized as one of “value contradiction,” as they navigate the delicate balance between recognizing the benefits and being acutely aware of the challenges inherent in integrating ICT into educational contexts. This stance showcases cautious optimism rooted in a thorough assessment of the multifaceted impact of ICT.

Cluster 4–Skeptical Conservative Parents: Represented by a sole father, this group constitutes a mere 1.4% of parents who maintain a more conservative and skeptical stance toward the educational value and potential risks associated with ICT. These parents assign the lowest ratings to both the positive and negative aspects of incorporating computers into education. Their standpoint reflects a cautious and critical viewpoint, characterized by reservations about the extent of ICT’s benefits within educational contexts.

Cluster 5–Pessimistic Believer Parents: Encompassing approximately 16.7% of parents, this cluster holds the viewpoint that ICT brings forth various negative effects and harbors reservations about its educational value. Within this group, skepticism is prevalent concerning the potential downsides of ICT. These parents exhibit a perspective that is marked by a blend of pessimism and belief, as they acknowledge the negative aspects of ICT’s influence on education while also harboring a certain degree of conviction in their assessments.

Overall, five clustered parent groups displayed distinctive differences in terms of their conceptions of children’s ICT use. These clusters highlight the complex web of parental attitudes toward children’s ICT use, reflecting the interplay between potential benefits and risks. A prominent finding has emerged, revealing that only a mere 23.5% of parents firmly believe in the positive impact of ICT. This particular group downplayed minor negative consequences, asserting that the substantial advantages of technology outweigh any potential drawbacks. In contrast, the majority of parents demonstrated varying degrees of uncertainty regarding the educational value of ICT. They held reservations due to the perceived negative effects associated with its usage, even as their levels of positive sentiment varied.

### Family SES as a predictor of ICT use in early childhood

We examined the impact of family SES on children’s ICT use within a subset of parents with children (*n* = 336) with computer experience and (*n* = 219) with smartphone experience, all younger than 8. Initially, concerns regarding multicollinearity were alleviated, given that the variance inflation factor (VIF) remained below 10, and tolerance surpassed 0.20, as recommended by Field (2009). A moderate correlation (*r* = 0.31) emerged between parents’ education and monthly household income. The results revealed limited explanatory power of family SES on the actual age and frequency of computer use. However, parents with higher educational degrees tended to have children who started using computers at an earlier age and used them less frequently. For smartphone use, family SES showed no predictive relationship with appropriate age, actual age, or frequency. Nevertheless, parents with higher educational degrees tended to introduce smartphones at a younger age, with their children using them less frequently than those of parents with lower educational degrees. Overall, family SES, particularly parents’ educational levels, can influence children’s ICT use, with a more significant impact observed for computer use than for smartphone use (Table 6).

**TABLE 6:** Multivariate regression analysis of family SES and ICT use by children under the age of 8.

Criterion predictor	Appropriate age			Actual age			Frequency		
	**b**	**β**	***t***	**b**	**β**	***t***	**b**	**β**	***t***
**Using a computer**									
Education	0.09	0.02	0.34	−0.05	−0.11	−1.71	−0.13	−0.18	−3.00**
Monthly household income	−0.10	−0.03	−0.44	−0.04	−0.09	−1.50	0.03	0.05	0.45
*R*	*R* = 0.06, *R*^2^ = 0.003			*R* = 0.16, *R*^2^ = 0.03			*R* = 0.17, *R*^2^ = 0.03		
*F*	*F* = 0.44			*F* = 3.88*			*F* = 4.50*		
**Using a smartphone**									
Education	−0.03	−0.03	−0.44	−0.01	−0.03	−0.36	−0.12	−0.15	−2.10
Monthly household income	0.06	0.09	1.12	−0.04	−0.09	−1.15	0.08	0.12	1.60
*R*	*R* = 0.08, *R*^2^ = 0.01			*R* = 0.10, *R*^2^ = 0.01			*R* = 0.16, *R*^2^ = 0.03		
*F*	*F* = 0.53			*F* = 0.92			*F* = 2.71		

*p < 0.05, **p < 0.01.

### Parental attitudes as a predictor of regulating young children’s ICT use

Building on Vandewater et al. (2005) study, our research explored whether parental attitudes toward children’s ICT use predict their regulation of ICT use. The findings in Table 7 demonstrate a significant relationship between parental attitudes and the regulation of ICT use. Parents with more positive views on the effects of children’s ICT use tended to have more permissive regulations. Conversely, parents expressing greater concerns about potential negative effects imposed stricter regulations on their children’s ICT use.

**TABLE 7:** Multivariate regression analysis of parental attitudes and regulations.

Criterion predictor	Parental regulation of young children’s ICT use					
	**Computer**			**Smartphone**		
	**b**	**β**	***t***	**b**	**β**	***t***
Positive effect	−0.21	−0.30	−7.33***	−0.12	−0.15	−3.43***
Negative effect	0.09	0.14	3.30***	0.08	0.10	2.37*
	*R* = 0.37, *R*^2^ = 0.14			*R* = 0.21, *R*^2^ = 0.05		
	*F* = 49.85***			*F* = 14.17***		

*p < 0.05, ***p < 0.001.

H_2_ was partially supported.

## Discussion

### The shift in young children’s media consumption

While television remains the primary medium through which young children consume media, the potential for increased use of ICT by this age group cannot be overlooked. The results of this study, consistent with previous findings, indicate that over half of young children have experience with ICT (Bedford et al., 2016). Although this study reveals that the number of children using computers is approximately 20% higher than that using smartphones, the ages at which children begin using computers or smartphones appear to be quite similar. Regardless of the device, similar to the Kuo (2022) study, approximately 70% of experienced young children in this study were exposed to computers or smartphones during their kindergarten stage. This trend may be attributed to the growing integration of ICT in educational settings. With the portability of ICT devices, many researchers and educators advocate for their effective use to harness the tremendous educational potential they offer to preschool children (Kim and Smith, 2017). Furthermore, there is a wide range of digital learning games, materials, and mobile apps specifically designed for preschool children, offering a blend of education and entertainment. Many parents choose to purchase online resources to support their children in developing the essential math and language skills required by preschool curricula (Papadakis et al., 2019; Dias and Brita, 2021). This parental behavior helps explain why most children come into contact with technology products during their preschool years.

The combination of increased accessibility, the educational potential of digital resources, and parental investment contributes to the early exposure of young children to ICT (Papadakis et al., 2019). This trend reflects the evolving landscape of early childhood education and highlights the role of technology in fostering early learning experiences. Among young children with experience in ICT use, the early introduction of smartphones compared to computers may be attributed to their convenience. Smartphones are typically readily available and easily carried, and parents can easily hand them to their children to use. However, the omnipresence of these mobile devices also poses potential risks, such as the risk of addiction or excessive reliance (Dias and Brita, 2021; Gür and Türel, 2022), which are concerns frequently expressed by parents.

### Parental companionship in young children’s ICT exploration

The study reveals that less than 40.66% of parents reported always accompanying their child during ICT use. In other words, approximately 60% of parents did not consistently participate in their children’s technology usage. This finding aligns with a previous study conducted by Common Sense Media (Rideout et al., 2020). However, parental companionship plays a crucial role in the cognitive development of young children when using ICT. Wood et al. (2016) research demonstrated that parental companionship, characterized by various forms of support, can scaffold young children’s learning during ICT use. This support can take the form of physical assistance (e.g., pointing to the screen), verbal guidance (e.g., reading aloud, commenting, providing hints and examples, and offering direct instruction), emotional support (e.g., praise and encouragement), and physical-emotional support (e.g., cuddling or hugging the child).

Flynn and Richert (2015) also found that parental support contributes to children’s deeper understanding of ICT content, thereby promoting cognitive learning. In essence, the active supervision and guidance provided by parents during children’s ICT use are essential for maximizing the benefits of ICT in educational settings (Kim and Smith, 2017; Hatlevik et al., 2018). Parents should employ effective strategies to regulate their children’s ICT usage and mitigate potential risks associated with the internet. Additionally, parents’ attitudes toward their children’s internet use are crucial in shaping their online behavior. By implementing appropriate strategies and maintaining positive attitudes, parents can promote responsible ICT use and ensure a safer online experience for their children.

Consistent with the aforementioned findings, the majority of parents in this study perceived cognitive learning as the most meaningful and significant benefit of ICT use. Furthermore, the more parents recognize the positive effects of ICT use, such as access to rich and diverse content, the more lenient they are in regulating their young children’s ICT use (Dias and Brita, 2021; Ko and Park, 2023). However, in the absence of proper parental guidance, the cognitive learning outcomes associated with ICT use may be compromised. Young children cannot derive the full cognitive benefits from ICT without the support provided by their parents during ICT learning activities. The lack of parental support may even contribute to negative effects, such as addiction (Gür and Türel, 2022), which parents are particularly concerned about. Consequently, this may result in a dual loss for young children in their ICT use, where they spend excessive time on ICT without reaping the positive cognitive learning effects.

### Perceived vs. actual: age discrepancy in children’s ICT initiation

This study reveals a significant discrepancy between parents’ perceptions of the appropriate age for children to start using ICT and the actual age at which their children begin using these technologies. Regarding the suitable age for children to start using computers, 48.59% of parents considered 6 years old or later, and 58.52% considered smartphones to be suitable at this same age. However, over 80% of parents reported that their children had initial exposure to computers and smartphones before the age of six. Evidently, there exists a disparity between the perceived and actual age of children’s ICT use. It is worth examining the reasons for this disparity.

One potential explanation for this divergence could be rooted in parents’ ambivalent attitudes toward their children’s engagement with ICT. The study of Papadakis et al. (2019) also highlighted the challenge parents face in delineating the boundary between the constructive utilization of digital technologies that facilitate children’s learning and the potentially detrimental usage of these devices. On the one hand, parents may acknowledge the prospective advantages of technology in enhancing education and cultivating skills. Conversely, they might also hold reservations concerning the potential adverse consequences of excessive screen time and its potential impact on early childhood development. Consequently, parents find themselves negotiating a delicate equilibrium between perceived benefits and potential drawbacks of technology for their children. They may aspire to harness the educational prospects presented by ICT while concurrently remaining vigilant about potential pitfalls. Attaining this equilibrium might pose a significant challenge for parents, as they endeavor to make well-informed decisions about their children’s exposure to technology.

Another contributing factor could be the intricate balancing act parents engage in, as they juggle their work commitments, household duties, and various other pressures. In a recent study by Ko and Park (2023), it was revealed that primary caregivers are influenced by the weight of parenting responsibilities and stress, leading them to permit their children’s smartphone usage. The authors suggest that smartphone use might serve to enhance children’s cooperation and provide caregivers with moments of relief. However, this respite is short-lived, as caregivers swiftly encounter negative emotions and heightened concerns about the potential adverse effects of smartphones on their children. As a result, they find themselves oscillating between granting permission and imposing restrictions on their children’s smartphone usage.

The findings of this study underscore the intricate nature of parents’ perspectives on technology. These viewpoints are not solely polarized into positive or negative realms; rather, they are shaped by the intricate interplay of challenges and opportunities that the digital age presents. In light of these complexities, it is important for the broader conversation to move beyond the binary question of whether children should engage with digital technologies. Instead, emphasis should be placed on maximizing the potential benefits that digital technology can offer for children’s learning and holistic development (Goulding et al., 2018; Demetriou and Nikiforidou, 2019; Swider-Cios et al., 2023).

### Gendered perspectives: contrasting stances of fathers and mothers on children’s ICT usage

Moreover, the discrepancy in fathers’ greater inclination toward embracing negative viewpoints compared to mothers provides an illuminating glimpse into their distinct outlooks concerning their children’s engagement with ICT. In contrast to the findings of the Lim and Soon (2010) study, which indicated that mothers perceived their partners as imposing overly stringent restrictions on their children’s ICT usage, the fathers in this current study exhibited a more pessimistic stance toward the potential benefits of ICT for their children. This variance could be attributed to the methodology employed by Lim and Soon, who indirectly discerned the differing viewpoints between spouses via maternal opinions rather than directly eliciting paternal perspectives.

Furthermore, a saying from the renowned Chinese “Three Character Classic” states: “If the son is not taught, the father is at fault.” Within the context of parenting, traditional Chinese norms assign fathers a significant role in enforcing discipline, even though mothers generally assume primary caregiving responsibilities. This proclivity is reinforced by the backdrop of the traditional Chinese cultural paradigm of “strict fathers and loving mothers.” Consequently, fathers often adopt a more stringent approach to their children’s upbringing due to their inherent sense of accountability for any shortcomings and their commitment to upholding a suitably rigorous paternal role (Xing et al., 2019). This tendency could elucidate why fathers are more prone to adopt a critical perspective regarding their children’s utilization of technology in comparison to mothers.

### Clustered perspectives: diverse attitudes toward children’s ICT use

Significantly, the study employed cluster analysis to categorize parents’ attitudes toward children’s ICT usage into five distinct clusters, encompassing a range of perspectives from balanced and positive views to value emphasis, conservative, and negative doubts. The presence of five distinct clusters highlights the rich diversity of perspectives among parents. The largest group, “Balanced Insightful Parents” (38.62%), maintains a well-rounded perspective, recognizing both positive and negative impacts of computers on children’s development. “Optimistic Believer Parents” (23.23%) express strong confidence in computers’ positive effects, while “Value Contradictor Parents” (19.87%) prioritize their benefits while acknowledging negatives. A small “Skeptical Conservative Parents” group (1.5%) exhibits caution toward both aspects, and “Pessimistic Believer Parents” (16.83%) express complex doubts about educational value and negative impacts.

In essence, these clusters epitomize the wide spectrum of parental attitudes, underscoring the necessity for tailored strategies to guide children’s responsible and constructive use of technology. As highlighted by Dias and Brita (2021), attempting to condense parental guidance in the realm of digital media into two dichotomous categories—restriction and empowerment—falls short in accurately capturing the nuanced approaches adopted within individual families. The intricate and dynamic nature of parental mediation defies such an oversimplified classification. With this, future studies should extend beyond a mere categorization of parental viewpoints. The diversity of attitudes underscores the intricate relationship between human perception and technology and raises questions about how these perspectives influence parenting practices, educational policies, and the design of technological tools for children. It would be intriguing to delve further into the specifics of each cluster to tailor effective strategies aimed at promoting responsible ICT usage among young children. This exploration could elucidate targeted approaches that address the distinct concerns and perspectives within each cluster, ultimately fostering a more informed and balanced approach to children’s engagement with ICT.

### Parental education as a key predictor of young children’s ICT use

Finally, in relation to predicting young children’s use of ICT based on parental SES, the findings of this study suggest that parental education level is a more reliable predictor than total monthly family income. This observation can be attributed to the growing prevalence of ICT and internet access. A 2013 survey by Common Sense Media revealed that the percentage of lower-income children who have used smartphones increased significantly from 22% in 2011 to 65% in 2013. This indicates that the digital divide across socioeconomic classes is narrowing (Kim and Smith, 2017). In today’s technologically advanced world, access to ICT is becoming more ubiquitous, irrespective of total family income. Consequently, using total family income as a predictor for young children’s ICT use may not be as effective as it once was.

Conversely, the study highlights that parental education level plays a more significant role in predicting young children’s ICT usage. Higher levels of parental education are associated with early exposure of young children to ICT, but it also results in more stringent regulation of its usage (Livingstone, 2007; Rideout, 2017). This suggests that parents with higher education levels tend to strike a better balance between recognizing the positive value and potential negative impacts of ICT on their children. They adopt an open and accepting approach to their children’s ICT usage while simultaneously controlling the time spent on ICT to avoid addiction and dependency. This enables children to effectively harness ICT for educational and learning purposes.

Echoing the previous perspectives that emphasize maximizing the potential benefits of ICT, this finding elucidates the changing dynamics of predicting young children’s ICT use and underscores the importance of parental education in guiding their children’s technology usage in today’s technologically advanced world. The lack of sufficient parental guidance in navigating the realm of ICT may lead to negative consequences, hindering children from attaining the desired benefits while simultaneously increasing the risks of adverse effects (Swider-Cios et al., 2023). However, a significant challenge arises from the realization that parents may lack the necessary guidance and skills to facilitate their children’s appropriate and responsible use of ICT (Radesky et al., 2016; Arumugam et al., 2021). Therefore, it becomes paramount to raise parental awareness and understanding of the appropriate use of ICT. By doing so, parents can effectively accompany and guide their young children, ensuring a balanced and advantageous ICT experience that aligns with their developmental needs. This approach will contribute to fostering a healthy and constructive relationship between young children and technology, promoting their overall growth and wellbeing.

## Conclusion

The study revolves around the exploration of ICT usage among young children and the corresponding attitudes of their parents. A pivotal discovery highlights a significant disjunction between the age parents ideally envision for their children to commence ICT usage and the actual age at which most children embark on this digital journey. This disparity exposes a contrast between parental aspirations and the pragmatic initiation of ICT exposure. Furthermore, a captivating finding was that fathers exhibited a conspicuously higher inclination toward accepting negative viewpoints than mothers. The study employed cluster analysis to categorize parents’ attitudes toward children’s ICT usage into five distinct clusters, reflecting a spectrum of perspectives from balanced and optimistic views to value emphasis, conservative, and negative doubts. This juxtaposition underscores the multifaceted nature of parental attitudes toward technology, molded by the opportunities and challenges inherent in the digital age. This intricate perspective necessitates a thorough comprehension, recognizing the nuanced layers embedded within parents’ viewpoints on ICT. This complex interplay of parental attitudes underscores the imperative for tailored approaches to digital education and guidance. Such strategies should aptly acknowledge and harness the array of viewpoints existing within families.

### Suggestions for future study

The findings of this study serve as a compelling call for ongoing research in a rapidly evolving technological landscape. They underscore the pressing need to align educational initiatives and guidelines with the complex realities that both parents and children encounter in the digital era. Moving forward, it is essential for research to explore the origins of gender-based disparities in parental attitudes toward ICT and subsequently investigate how these distinct ICT profiles among parents correlate with their strategies for regulating technology usage.

Employing qualitative research methods in these future investigations could reveal the intricate motivations and rationales that shape the perspectives of both fathers and mothers. A deeper exploration into the sociocultural, psychological, and societal influences that impact these viewpoints holds the potential to provide a more profound understanding of this multifaceted phenomenon. By delving into the underlying factors that contribute to these distinct parental attitudes, researchers can illuminate the broader societal dynamics that influence the integration of technology into family life. This knowledge would not only inform more effective educational strategies but also contribute to a comprehensive framework for fostering healthy and responsible technology use among the younger generation.

### Limitations

This study provides valuable insights into parents’ attitudes and the role of SES in shaping children’s media usage. However, there are certain limitations that should be accounted for when interpreting the results. First, the use of a cross-sectional research design in Taiwan means that caution should be exercised in drawing causal associations and generalizing the findings to other regions or cultural contexts. Conducting longitudinal studies that track participants over an extended period could yield more robust evidence of causal relationships and changes in media usage patterns over time. Importantly, the study solely focused on young children under 8 years old, which offers a valuable understanding of early childhood media usage. However, this approach leaves out the media habits of older children and adolescents. Expanding the study to include a broader age range would provide a more comprehensive perspective on how media usage patterns evolve as children age. Furthermore, including older age groups would enable a broader examination of the influence of parental attitudes and SES on media usage across developmental stages. Another limitation is that the study did not measure parents’ digital skills, which could offer additional insights into parent–child interactions with technology. Future research should consider investigating the impact of parents’ digital skills on children’s technology experiences. By doing so, a more comprehensive understanding of this subject can be fostered within the research community, leading to the development of educational initiatives and guidelines that effectively support parents and their children in navigating the digital era.

## Data availability statement

The raw data supporting the conclusions of this article will be made available by the authors, without undue reservation.

## Ethics statement

Ethical review and approval was not required for the study on human participants in accordance with the local legislation and institutional requirements. The patients/participants provided their written informed consent to participate in this study.

## Author contributions

YL, SY, KC, and HL contributed to the conception and design of this study, the analysis of the results, and writing of the manuscript. All authors contributed to the article and approved the submitted version.
